# Rabies Vaccination in Dogs in Laos: Owner Knowledge and Serological Status of Dogs

**DOI:** 10.3390/pathogens11010069

**Published:** 2022-01-06

**Authors:** Lovisa Velander, Johanna Fogelberg, Vannaphone Putthana, Amphone Keosengthong, Johanna Frida Lindahl

**Affiliations:** 1 Department of Clinical Sciences, Swedish University of Agricultural Sciences, 75007 Uppsala, Sweden; Lovisa@velander.se (L.V.); johanna.m.fogelberg@hotmail.com (J.F.); 2 Department of Veterinary Medicine, Faculty of Agriculture, Nabong Campus, National University of Laos, Vientiane 0100, Laos; v.phouthana@nuol.edu.la (V.P.); keosengthong1990@gmail.com (A.K.); 3 Department of Medical Biochemistry and Microbiology, Uppsala University, 75123 Uppsala, Sweden; 4 International Livestock Research Institute, Hanoi 100 000, Vietnam

**Keywords:** neglected tropical diseases, rabies virus, vaccination program, eradication, knowledge, attitudes and practices

## Abstract

Rabies is an infectious disease which is virtually 100% fatal. Humans are most often infected through the bite of an infected dog, and most cases could be prevented by vaccinating dogs. However, vaccination coverage is insufficient in most countries where canine rabies occurs endemically. This study conducted interviews and sampling of dogs in Laos to understand more about the barriers for vaccination and to evaluate the antibody status of dogs using a commercial ELISA. The study found that only 62% out of 359 dog owners knew what rabies was, and only 24% knew the disease could be fatal. Higher education was associated with higher knowledge scores. Only 56 out of 437 (13%) dogs had been rabies vaccinated according to their owner, and out of these dogs, only 34 (61%) had antibodies, and only 48% had adequate levels (above 0.5 IU/mL). However, 24% of the dogs with no known history of vaccination had antibodies, indicating either exposure or vaccination in the past without the owner’s awareness. In conclusion, this study indicates that there is a low level of knowledge about rabies, and that owner knowledge is not a good indicator of whether a dog is vaccinated or not.

## 1. Introduction

Rabies is caused by a lyssavirus in the family rhabdoviridae that can infect all mammals, and once symptoms appear it is nearly always fatal [1,2,3]. Rabies is also a disease that is 100% preventable through vaccination [4]. Despite that, it is still endemic in many regions in the world, where most cases of human rabies are seen in Africa and Asia [5,6]. Globally, canine rabies is responsible for more human deaths than any other zoonotic disease [7], and the vast majority of the estimated 59,000 human deaths [8] worldwide are the result of bites from rabid dogs, with most deaths occurring in Asia [4,9,10] and children being most affected. Since dogs are the main reservoir and source of infection for humans, vaccination of dogs is recognized as the most cost-effective and permanent solution to rabies prevention [11,12]. 

Some areas in the world are considered free of dog-mediated rabies [4,5]. For a country to be able to become free of canine rabies it is recommended that 70% of the dog population, both owned and stray, has an adequate immunization against rabies virus [13,14]. Parenteral vaccination remains the most reliable method for ensuring adequate immune response, but the difficulties in reaching all stray dogs, as well as the high replacement rate of the population, may result in a decline in the vaccination coverage between campaigns to below 20–45%, which is insufficient to control rabies [15].

One country that is endemic of canine rabies is Lao People’s Democratic Republic (PDR) [16]. Laos is located in Southeast Asia with around 7.2 million inhabitants, and between the years of 2012–2017, there have been 33 reported cases of human rabies deaths in the country [17]; however, this is likely to be much larger due to a great amount of unreported cases. Laos considers rabies as a top-five priority disease and every year is treating more than 8000 people with post-exposure prophylaxis [18]. Worldwide, dogs are the main transmitter of rabies virus (RABV) to humans [19], and therefore, the aim of this study was to investigate the knowledge regarding rabies among dog owners in Vientiane Prefecture, where most cases of rabies in the country is reported [16], and to investigate the vaccination status and protection level against RABV. 

## 2. Results

**Figure 1:**
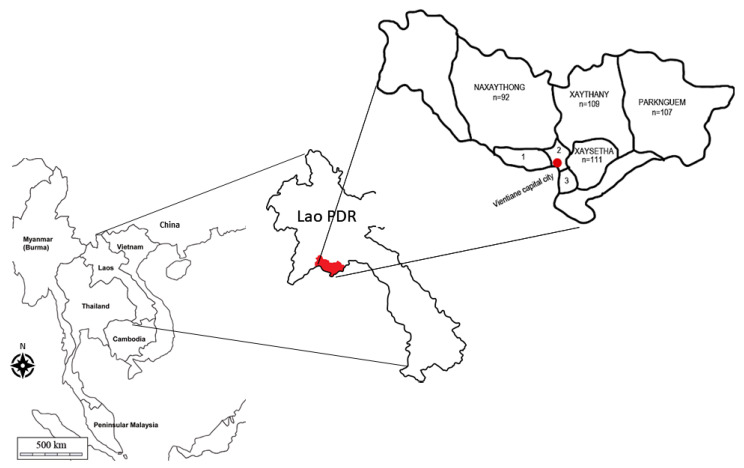
Map of Vientiane prefecture in Lao People’s Democratic Republic (PDR) showing the number of samples in each district. 1: Sikhottabong (n = 9), 2: Chanthabouly (n = 6), 3: Sisattanak (n = 3).

The survey was conducted in connection with vaccination campaigns where dog owners were offered rabies vaccines. The visiting dog owners were informed about the study and asked for their consent to participate. A total of 359 dog owners and 437 dogs from seven districts (Figure 1) participated. 

### 2.1. Questionnaire Results

The age of the dogs varied from 2 months to 12 years, which results in an average age of approximately 2 years. Most, 96.9%, used the dog as a guard dog and 13.6% as company. It was common to keep dogs loose, with 68.2% spending all their time outside loose. Nearly 75% had received the dog as a gift.

**Figure 2:**
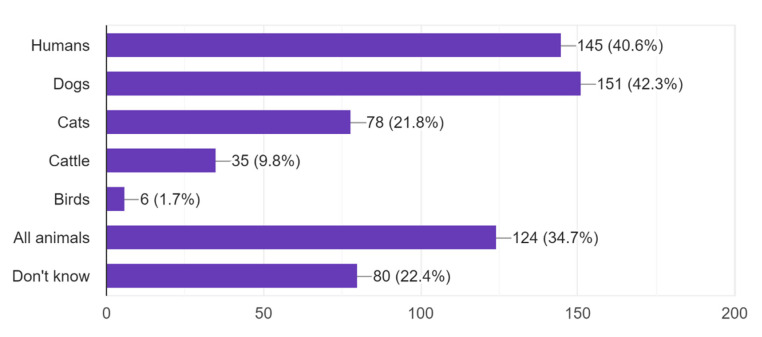
Number of participants that answered who can get rabies. More than one alternative could be chosen.

The result showed that 62.4% of the participants knew what rabies is, and 59% knew that rabies is transmitted by bites. In total, 352 people answered the question whether rabies can be fatal or not. Out of which 24.1% answered “Yes”, 17.9% answered “No” and 58% answered “Don’t know”. When asked who can get rabies, 357 respondents answered, with more than 40% answering dogs or humans (Figure 2).

**Figure 3:**
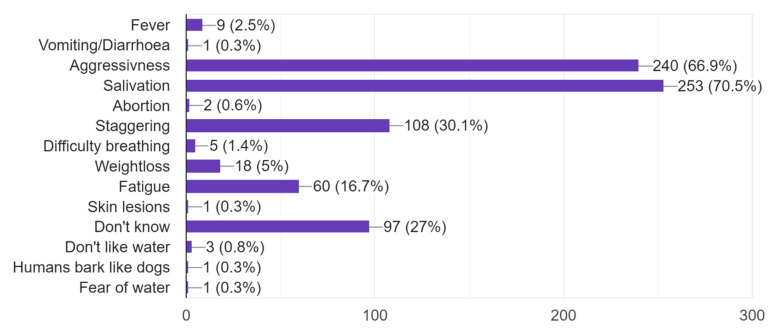
Number of participants with their answer to mention symptoms of rabies in humans and dogs. More than one alternative could be chosen.

When asked to mention which symptoms rabies can give an infected person or dog, all 359 respondents answered, of which 27% said that they did not know (Figure 3). The knowledge about symptoms of rabies was quite high. In total, 70.5% answered salivation and 66.9% answered aggression; however, only 30.1% said that staggering is a symptom of rabies. 

Over two-thirds (68.2%) knew that there is a vaccine against rabies. The main source of knowledge about rabies was from a friend or someone from the older generation (58.6%). In total, 19.7% of the respondents answered that they received information about rabies from government rabies vaccination campaigns. In total, 18.3% had received information from the veterinarian, 10.7% had received information through school and 15.5% had knowledge about the disease because they had seen a rabies case. Out of the 38 people that said that some knowledge came from school, five had primary education, one had gone to classes 5–10, 14 had gone to higher secondary school and 18 had a graduation or above.

**Figure 4:**
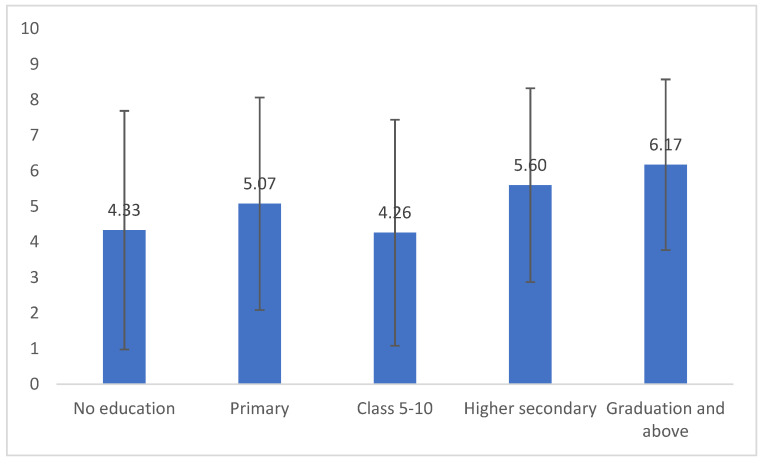
Average total knowledge score about rabies in each level of education.

The average knowledge score of all participants was 5.23, and the median score was 6, with 10 as the highest possible score. When investigating if there was a difference in knowledge score for the different educational groups, the results showed that the difference in mean value between the educational groups was statistically significant (*p* value = 0.001) (Figure 4). 

The average knowledge score for participants that had vaccinated their dog previously was 5.96 and for those who had not vaccinated their dog was 4.92. The average knowledge score for the participants who did not know if their dog had been previously vaccinated was 3.67. When testing whether there was a significant difference in average scores between the categories of vaccination status, the result showed that the difference in mean value was statistically significant (*p* value = 0.004). 

### 2.2. Serological Analyses and Vaccination Status

In total, 124 dogs had detectable antibodies against rabies, but only 57 had high enough antibody levels according to the ELISA test (above 0.5 IU/mL) to be considered to have an adequate antibody response and thus likely be protected. Out of the 56 dogs where the owner stated that the dog had been vaccinated against rabies, 34 dogs (60.71%) had detectable antibodies against rabies virus (Table 1). Compared to the dogs where the owner stated that the dog had not been vaccinated against rabies (n = 375), 89 dogs (23.73%) had detectable RABV neutralizing antibodies (Nab), making it significantly lower than the dogs reported to be previously vaccinated (*p* < 0.001).

When instead investigating the protection level among dogs, where the owner stated that the dog had been vaccinated against rabies before (n = 56), only 27 dogs (48.2%) had adequate antibody response, significantly higher (*p* < 0.001) than where the owner stated that the dog had never been vaccinated against rabies (n = 370), out of which 30 dogs (8.1%) had adequate antibody response (Table 1).

There was a significantly higher percentage of dogs with an adequate response in the districts closer to the capital (Xaysetha 18.27% and Xaytany 14.02%), compared to the districts further away (Parknguem 7.48% and Naxaythong 5.43%) (*p* = 0.015) (Table 2). Seropositive dogs were significantly older (3.5 years, standard deviation (SD) 2.8) than seronegative dogs (1.9 years, SD 2.2) (*p* < 0.001) (Table 3). 

### 2.3. Time Impact on Immune Status

When investigating time impact on immunization, the owners were asked when the dog last was vaccinated against rabies. Out of the dogs where the owner stated it had been vaccinated against rabies (n = 56), 12 dogs had last been vaccinated more than one year before the survey (21.43%) and 44 dogs had been vaccinated within the last year (78.57%). 

When comparing immunization between the two groups, 7 out of the 12 dogs (58.33%), who had last been vaccinated over one year before the survey, had detectable antibodies and 27 out of the 44 dogs (61.36%), who had been vaccinated within the last year, were positive for antibodies (Figure 1, *p* < 0.001). When instead looking at protection level, 5 of the 12 dogs (41.67%) that had been vaccinated over one year before the survey had adequate antibody response, and 22 of the 44 dogs (50%) that had been vaccinated within one year of the survey had adequate antibody response (*p* < 0.001) (Table 2). 

The odds of a dog having detectable antibodies against rabies was significantly higher for dogs who had been vaccinated against rabies over one year before sampling (odds ratio (OR) 5.8, *p* = 0.002) and for dogs who has been vaccinated within one year prior to sampling (OR 4.6, *p* < 0.001) compared to dogs who has never been vaccinated against rabies.

### 2.4. Reasons for Not Vaccinating Dogs

To achieve an understanding of why dog owners had not vaccinated their dog against rabies previously if they know that there is a vaccine, an open question was asked to a subset of participants: 14 people in Naxaythong, 3 people in Parknguem, 8 in Xaysetha and 5 in Xaytany. The majority answered that they had no time, or there was no veterinarian to give the vaccine, that the clinic was too far away, or that it was too expensive. A few additional reasons given were that the dog always became sick after an injection, that their dog was never angry towards other people and therefore did not need rabies vaccine, and that it was believed that one previous vaccination protected the dog from rabies its whole life. 

## 3. Discussion

This project investigated knowledge of rabies in dog owners in Laos, the perceived vaccination status according to the owners, as well as the actual antibody status of the dogs. Our results showed that the proportion of positive dogs for RABV is not sufficient to prevent human cases of dog-mediated rabies in Vientiane Prefecture, Laos, since only 28.32% of sampled dogs had detectable RABV NAb, which is shown to be insufficient to eliminate rabies. This study is not representative of the entire country, since we purposively selected the area where most rabies in dogs had been reported, and districts with previously reported cases. The four districts Naxaythong, Parknguem, Xaysetha and Xaytany had 22, 2, 43, and 105 dog rabies cases, respectively, reported 2010–2016 [16]. 

There is a global strategic plan “Zero by 30”, where the goal is that there should be zero dog-mediated human deaths of rabies by 2030. The plan is put together by United Against Rabies, a collaboration between the World Health Organization (WHO), Food and Agriculture Organization of the United Nations (FAO), World Organization for Animal Health (OIE) and Global Alliance for Rabies Control (GARC) [20,21]. There is a Stepwise Approach to Rabies Elimination (SARE) by the Rabies Alliance, which gives countries different scores depending on how far the country has reached, and here Laos is judged to have a SARE score 0.5 out of 5 [18]. There are occasional campaigns in Laos to increase awareness of rabies [18]. To interrupt transmission of canine rabies, and thereby eliminating the risk of human cases, the recommendations are that 70% of the dog population is vaccinated [22,23]. However, upholding this level of immunity is problematic. Dog population turnover is often high in Southeast Asia due to many diseases and consumption of dogs for food [24], and in neighbouring Cambodia, the proportion of dogs surviving 24 months in two provinces were 52% and 34%, separately, and there were high dog to human ratios, with one dog for every 3.3-3.8 humans [25]. This is a higher ration than in Nigeria and Thailand, where the dog to human ratio were 1:4.1 and 1:4.6, respectively [26,27]. Based on the dog and human population in Sweden 2012, the ratio of Sweden was 1:13.1, as a comparison [28]. 

In this study, over 60% of interviewed participants claimed to know what rabies is; however, it does not necessarily mean that the participants actually have that knowledge, since this may also include people that have just heard about the term. However, 57.9% said that they know what rabies is and could also mention who can be infected, which is rather close to the percentage of people claiming to know what rabies is, thus the number could very well be correct. It is possible that this percentage is closer to the actual number of people knowing what rabies is. When asked an open-ended question, 40.9% mentioned rabies when asked to state an illness that dogs can transmit to humans. The people that had chosen to vaccinate their dogs before had a higher knowledge score. The fact that more dogs in the districts closer to the capital were vaccinated could be correlated with higher education and knowledge in the city. 

Since bites by rabid dogs are by far the most common cause of human rabies, information about transmission should be well spread in endemic countries [17]. In the present study, 59% of the participants knew that rabies is transmitted by bites. If this is representative for the entire Laotian population, this means that two-fifths would not know that you can get rabies if bitten by a dog and might not seek medical care. The incidence of dog bites in Cambodia was shown to be high, at 2.3–3.1% yearly incidence [25], and this is likely similar to the situation in Laos. If all dog owners that know about the vaccine in Lao PDR (68.2%) actually vaccinated their dog against rabies, it would almost reach the vaccination coverage goal of “Zero by 2030” which would have a positive effect on the number of human rabies cases. 

However, our results also showed that there are discrepancies between dog owners’ information regarding previous rabies vaccinations and the results of detectable antibodies of the dogs. Only 50% of dogs vaccinated within a year had adequate antibody levels. Interestingly, 8% of dogs said never to have been vaccinated also had antibody levels exceeding 0.5 IU/mL, and 23.7% had antibodies. This could be due to owners not understanding or knowing that the dogs were vaccinated, but there has also been a number of other studies showing unvaccinated dogs with antibodies exceeding 0.5 IU/mL [29], raising the question if it may be possible for dogs to be exposed to rabies and survive. 

There are multiple factors influencing vaccination coverage, including the owners’ willingness to vaccinate. In a study performed by Castillo-Neyra et al. [30], the owners’ willingness to participate in vaccination campaigns decreased in proportion to the distance from the owners’ house to the place of vaccination campaigns, proving the importance of conducting vaccination campaigns in close proximity to the target population. In this study, a subset of the participants who said that they were aware about the vaccine were asked why they had not vaccinated before, and the main reasons stated were the distance to a vaccination clinic and availability of a veterinarian. Therefore, to further improve the vaccination status among dogs in Vientiane Prefecture, it would be necessary to conduct vaccination campaigns that are accessible even to people living in districts further away from Vientiane capital city, and to increase the general availability to veterinarians and vaccinations.

But there are also aspects concerning the vaccine and the dog being vaccinated. Factors affecting vaccines may include extreme temperatures during storage [31], unless provisions are taken for cooling the vaccines. Since Laos is a country with mainly tropical climate and high average temperature, the effect of vaccination can be decreased by inadequate handling of vaccines due to deficient cold chain, as the vaccine may be deteriorated.

Some factors may affect the immune response after vaccination. In humans it has been shown that chronically ill or immunocompromised individuals might not seroconvert as efficiently as healthy individuals [32,33], and this is likely also the case for dogs. Similarly, exposure to different mycotoxins can negatively affect the response after vaccination in animals [34,35], which may also be true for the dogs in this study, as Southeast Asia have high prevalence of contaminated food and feed [36,37,38], even though Laos has been little studied. Difference in immunization between dogs can also be explained by the usage of different brands of rabies vaccines [39]. A study in Sweden found that the brand of vaccine, the size of the dog, as well as the age could affect the chance of a dog reaching an adequate level of antibodies, but overall, more than 90% of dogs would reach 0.5 IU/mL after vaccination [40].

## 4. Materials and Methods

### 4.1. Study Design

The cross-sectional study was conducted in districts of Vientiane Prefecture, Laos (Figure 1), in September and October 2019. This is the part of Laos where most rabies cases are reported; 90.5% of all reported cases in 2010–2016 occurred in the Vientiane Prefecture [16]. Four districts with confirmed prior occurrence of canine rabies were selected purposively, in addition to participation in one vaccination campaign in the capital. Before data collection, the village chief agreed to allow the study in their village. The village chief received a paper about the study and was asked not to inform the dog owners about rabies until after the study in order not to influence the knowledge results. 

The data collection was conducted in connection with vaccination campaigns. All dogs were given rabies vaccine and ivermectin against endo- and ectoparasites. The village chief informed the villagers over the loudspeakers in the villages. Four days of the data collection a speaker was not available, and data collection was performed by visiting the dog owners’ homes. As the survey was conducted in association with campaigns, there was no pre-calculated sample size but all visitors to the clinics were asked for their interest to participate and included if they consented. Participants were thus not randomly selected and not representative of the entire area. 

### 4.2. Survey

The inclusion criteria for participants was that they owned dogs, and they signed an informed consent form before being allowed to participate in the study. A structured questionnaire was developed and discussed at the Faculty of Agriculture, National University of Lao (NUOL). The questions were thereafter modified to better suit the target group. The questionnaire was translated from English to Lao. The owners were asked questions about themselves, the dog and about rabies (see Supplementary Material S1).

### 4.3. Serological Analyses

The inclusion criteria for sampling dogs was that the dogs were older than three months, and showed no signs of aggression, for the safety of the personnel. If there were more dogs in one household, only two dogs were used in the study. After sampling, the owners received an information pamphlet regarding rabies that had been translated into Lao. 

All dogs participating in the study were wearing muzzles and handled by confident staff throughout the whole process of blood sampling. The blood was collected in serum blood collection tubes and centrifuged to receive serum. The serum was stored in a freezer at approximately -19 degrees Celsius for preservation until analysed. After being thawed and brought to room temperature, the serum samples were analysed for rabies virus antibodies using the BioPro Rabies ELISA Ab kit (Prague, Czech Republic), a test that has been shown to correlate well with the gold standard test [41]. A total of 283 of the 437 samples (64.76%) were run in duplicates to ensure the authenticity of the test. The mean value was then calculated for results. Out of the duplicates, there were 14 samples (4.95%) with dubitable results, and they were therefore analysed again. 

The results were then calculated into percentage blockage of antibodies according to instructions and samples with a percentage equal or higher than 40% are considered positive for RABV NAb. Samples equal or higher than 70% blockage are considered to have an antibody level reaching up to, or higher than 0.5 IU/mL based on the FAVN test [42]. These samples are considered having an adequate antibody response and are likely representing protection against rabies. 

### 4.4. Data Entry and Analysis

The answers from the questionnaire were transferred into an Excel file. The knowledge about rabies was evaluated by a scoring system based on the questions asked. Question 6.1, 6.6 and 6.7 gave the respondent 1 point each if she/he answered “Yes”. Question 6.3 gave the respondent 1 point if she/he answered “Bites” or “Contact with dog saliva” and 2 points if both alternatives were chosen. Question 6.4 gave the respondent 1 point if she/he answered “Humans”, “Dogs” or “All animals”, 2 points if “Humans” and “Dogs” were chosen. Question 6.5 gave the respondent 1 point if she/he answered “Aggressiveness”, “Salivation” or “Staggering”, 2 points if two of the alternatives were chosen and 3 points if all three alternatives were chosen. No points were subtracted if the wrong answer had been chosen. The maximum points possible were 10.

Descriptive statistics were calculated. Investigating and comparing the results between different categories two tailed t-test, Pearson’s chi-squared test, logistic regression, and ANOVA was used. The *p*-value for statistical significance was set to <0.05 for all analyses. All statistical analyses were performed in Minitab except Pearson’s chi-squared test and logistic regression (logit) which were performed in the statistics software program STATA 14.2.

## 5. Conclusions

Our results show that the proportion of dogs positive for rabies antibodies is not nearly sufficient to prevent human cases of dog mediated rabies in Lao PDR. The results show important and interesting gaps in knowledge regarding rabies and its prevention. For example, the percentage of people knowing that rabies is fatal is relatively low which points out what information vaccination campaigns, veterinarians, doctors and other sources of information should focus on. In addition, the information from owners as to whether their dog was vaccinated or not turned out to be unreliable. Many dogs stated to be vaccinated did not have adequate antibody levels, which could give the owners a false sense of safety, and thus potentially more at risk as they may believe that post-exposure prophylaxis is not needed after a bite.

In conclusion, further work needs to be performed in Lao PDR regarding rabies elimination strategies, by engaging the society and educating about rabies and the importance of post exposure prophylaxis, as well as conducting mass dog rabies vaccination campaigns easily accessible to target populations. By doing this, the vaccination status and protection level among the canine population will be improved, and the goal of zero human deaths of canine rabies by 2030 could be achievable.

## Figures and Tables

**Table 1:** Rabies vaccination status of dogs according to dog owners in Laos, and the results from serological testing.

Vaccination Status According to Owner	Antibody Positive	Adequate Levels *
All dogs	124/437 (28.4%)	57/432 (13.2%)
Never vaccinated against rabies	89/375 (23.7%)	30/370 (8.1%)
Vaccinated against rabies	34/56 (60.7%)	27/56 (48.2%)
More than one year ago	7/12 (58.3%)	5/12 (41.7%)
Less than one year ago	27/44 (61.4%)	22/44 (50%)

* Adequate levels defined as having antibody levels exceeding 0.5 IU/mL.

**Table 2:** Proportion of dogs having detectable antibodies against rabies as well as adequate levels, compared between districts and time since vaccination.

Factor	Antibody Positive	Adequate Levels *
District		
Xaytany	29/109 (26.6%) ^c^	15/108 (13.9%) ^a,b^
Xaysetha	41/111 (36.9%) ^a,c^	20/107 (18.7%) ^b^
Parknguem	22/107 (20.6%) ^b^	8/107 (7.5%) ^a^
Naxaythong	21/92 (22.8%) ^b^	5/92 (5.4%) ^a^
Districts within Vientiane capital	11/18 (61.1%) ^a^	9/18 (50.0%)
Time from vaccination		
Vaccination within the last year	27/44 (61.4%)	22/44 (50.0%)
Vaccination over a year ago	7/12 (58.3%)	5/12 (41.7%)

* Adequate levels defined as having antibody levels exceeding 0.5 IU/mL ^a^, ^b^ etc.: proportions with the same superscript within column are not significantly different.

**Table 3:** Age of dogs depending on rabies vaccination status and antibody test results.

Status of Dog	Mean Age (years)	Standard Deviation	*p* Value
All dogs	2.3	2.5	
Never vaccinated against rabies	2.2	2.4	0.003
Vaccinated against rabies	3.2	2.7	
Rabies antibody positive	3.5	2.8	<0.001
Antibody negative	1.9	2.2	
Adequate levels of rabies antibodies	3.5	2.8	<0.001
Not adequate levels	2.2	2.4	

## Data Availability

Data will be made available from the corresponding author upon request.

## Supplementary Materials

The following are available online at https://www.mdpi.com/article/10.3390/pathogens11010069/s1, Supplementary Material S1: Questionnaire.
