# Supplementary material for: Rabies Vaccination in Dogs in Laos: Owner Knowledge and Serological Status of Dogs
Source: Pathogens. 2022 Jan 6;11(1):69. doi: 10.3390/pathogens11010069 (PMC8778959; doi:10.3390/pathogens11010069)
Supplement: Supplementary file 1 [file pathogens-11-00069-s001.zip › pathogens-1505314-supplementary.pdf]

Questionnaire

1. Which district or province do you live in:

2. Information about the dog owner

2.1 Gender: Female Male

2.2 Age (years):

2.3 Education level: No education Primary Class 5-10 Higher secondary Graduation and above

2.4 Are you the owner of the dog: Yes No

2.5 Do you consume dog meat: Yes No

2.6 If yes, how often: Every day Once a week Once a month Less often

2.7 If no, why not:

3. Dog information:

3.1 Age of the dog:

3.2 Breed:

3.3 The dogs' main use: Guard Company Meat Other:

3.4 Main caregiver: Adult in family Child in family Other:

3.5 Living situation: Only outside loose Only outside in a leash Only outside loose but in a fenced area
Only indoor Booth indoor and outside, when outside in a leash
Booth indoor and outside, when outside loose

3.6 How did you come to own the dog: Bought A gift Puppy from previous dog Other:

4. Health status of the dog:

4.1 Has the dog ever gotten vaccinated: Yes No (continue to 4.5) Don't know

4.2 How often does the dog get vaccinated: One time 1 time/year 1 time/3 years Other:

4.3 Against which illness:

4.4 How long ago was the dog vaccinated:

4.5 History of illness: Bite wounds Vomiting/diarrhoea Parasites Lameness
Other:

5. General questions:

5.1 Do you know if dogs can transmit diseases to humans: Yes No

5.2 If yes, which illness do you know of that dogs can transmit to humans:

6. Rabies:

6.1 Do you know what rabies is: Yes No

6.2 Do you know how rabies is transmitted: Yes No

6.3 If yes, how is rabies transmitted: Mosquitoes Faeces Bites Blood contact Food Contact with dog saliva

6.4 Who can get rabies: Humans Dogs Cats Cattle Birds All animals Don't know

6.5 Symptoms of rabies: Fever Vomiting/diarrhoea Aggressiveness Salivation Abortion Staggering Difficulty breathing Weight loss Fatigue Skin lesions Don't know

6.6 Can rabies be fatal: Yes No Don't know

6.7 Do you know if there is a vaccine against rabies: Yes No

6.8 Where does your knowledge about rabies come from: School Government rabies vaccination campaigns Newspaper/TV/radio Old generation/friend Other:
